# IGF-1 potentiates sensory innervation signalling by modulating the mitochondrial fission/fusion balance

**DOI:** 10.1038/srep43949

**Published:** 2017-03-09

**Authors:** Yuan Ding, Jianmin Li, Zhen Liu, Huaxiang Liu, Hao Li, Zhenzhong Li

**Affiliations:** 1 Department of Anatomy, Shandong University School of Medicine, Jinan 250012, China; 2 Department of Orthopaedics, Shandong University Qilu Hospital, Jinan 250012, China; 3 Department of Rheumatology, Shandong University Qilu Hospital, Jinan 250012, China

## Abstract

Restoring the contractile function of long-term denervated skeletal muscle (SKM) cells is difficult due to the long period of denervation, which causes a loss of contractility. Although sensory innervation is considered a promising protective approach, its effect is still restricted. In this study, we introduced insulin-like growth factor-1 (IGF-1) as an efficient protective agent and observed that IGF-1 potentiated the effects of sensory protection by preventing denervated muscle atrophy and improving the condition of denervated muscle cells *in vivo* and *in vitro*. IGF-1-induced Akt phosphorylation suppressed the mitochondrial outer-membrane protein Mul1 expression, which is a key step on preserving contractile property of sensory innervated SKM cells. Mul1 overexpression interfered with the balance between mitochondrial fusion and fission and was a key node for blocking the effects of IGF-1 that preserved the contractility of sensory-innervated SKM cells. Activation of AMP-activated protein kinase α (AMPKα), a mitochondrial downstream target, could block the effects of IGF-1. These data provide novel evidence that might be applied when searching for new approaches to improve the functional condition of long-term denervated SKM cells by increasing sensory protection using the IGF-1 signalling system to modulate the balance between mitochondrial fusion and fission.

A balance between protein synthesis and degradation maintains the healthy condition of normal skeletal muscle (SKM). When the balance is disrupted (e.g., during proximal peripheral nerve injury), muscle atrophy occurs, along with a series of serious clinical problems, especially motor dysfunction^1^,^2^. Poor motor function recovery manifests due to prolonged muscle denervation, which leads to a loss of regenerative potential, especially when nerve repair is delayed or when the injury is located at a proximal point^3^,^4^. Although previous studies have tried to unveil the mechanisms and candidate agents for promoting muscle function recovery, the specific mechanisms are still unclear. Additionally, no effective available agents have been shown to be responsible for muscle function recovery, except in situations when effective motor reinnervation was performed on denervated SKM within a short time frame.

Pharmacological intervention the most common method for preventing muscle atrophy and preserving the muscle contractility of denervated SKM before reinnervation has been established. However, in some severe situations (e.g., nerve injuries that occur at highly proximal locations or when many motor axons are destroyed), sensory protection, which reconnects the sensory nerves to the injured motor nerve stumps, has been shown to be a promising technique^5^,^6^. However, the donor sensory axons provided only a temporary or minor preservation of the denervated SKM unless the original motor nerves reached the denervated muscle in time. Hence, pharmacological intervention to enhance the efficacy of sensory protection is a promising approach. Insulin-like growth factor 1 (IGF-1), a factor with myogenic and neurotrophic activities, might be a candidate for pharmacological intervention because IGF-1 can induce a marked increase in muscle mass, reflecting better cellular conditions^7^,^8^,^9^,^10^. However, whether IGF-1 can potentiate sensory protection in denervated muscle should be further clarified.

Multiple biological effects of IGF-1 were produced by activating the phosphatidylinositol 3-kinase (PI3K)/Akt signalling pathway. The Akt signalling cascade regulates the growth of many tissues by suppressing protein degradation and improving synthesis^11^,^12^. Increased phosphorylation levels of Akt have induced SKM hypertrophy and have prevented denervated muscle atrophy^13^. Recently, it has been indicated that Akt activation maintains mitochondrial functions^14^,^15^. The energy-generating mitochondria have been the focus of studies on the preservation of cell function. Mitochondria are highly dynamic in morphology continuously undergo fusion and fission. It is well recognized that mitochondrial fusion maintains the function of mitochondria and clears pathogenic mitochondria, as well as maintains the precision of the mitochondrial genome. In contrast, mitochondrial fission leads to loss of mitochondria and results in muscle atrophy^16^,^17^,^18^. Maintaining mitochondrial homeostasis by modulating the balance between fusion and fission is critical for preserving function. Many lines of evidence indicate that the mitochondrial E3 ubiquitin protein ligase 1 (Mul1) is one of the factors responsible for regulating dynamic mitochondrial alterations. Mul1, also known as mitochondrial-anchored protein ligase (MAPL) or mitochondrial ubiquitin ligase activator of NF-κB (MULAN), is a mitochondrial outer-membrane protein with a cytosol-facing C-terminal RING-finger domain. Inhibition of Mul1 leads to an expansive and fused network of healthy mitochondria and prevents mitochondrial dysfunction, whereas overexpression of Mul1 induces fragmentation, depolarization, and finally, accumulation of pathologic mitochondria^19^,^20^. However, the actions of Mul1 in sensory-innervated SKM cells and its connection to the IGF-1 modulation of these cells are still unknown.

Another mechanism resulting in muscle atrophy is the activation of AMP-activated protein kinase α (AMPKα) induced by a decrease in energy production or an enhancement in mitochondrial fission. Mitochondrial fragmentation-activated AMPKα may induce the expression of atrophy-related genes, atrogin-1 and muscle RING finger 1 (MuRF1), which finally results in muscle mass loss^21^,^22^,^23^,^24^,^25^. Whether this mechanism is related to the upstream IGF-1 signalling is also still to be investigated.

The IGF-1 signalling system and its function in modulating the balance between mitochondrial fusion and fission may have a promising role in preserving the contractility of denervated SKM cells. The present study tests the following hypotheses: (1) IGF-1 may have more obvious effects on sensory-innervated SKM cells than on denervated SKM cells *in vivo* and *in vitro*; (2) IGF-1-induced Akt phosphorylation is a key step in the preservation of the contractility of sensory-innervated SKM cells; (3) IGF-1-induced Akt phosphorylation may suppress the expression of the mitochondrial outer-membrane protein Mul1, and overexpression of Mul1 may block the effects of IGF-1 on the preservation of the contractility of sensory-innervated SKM cells; (4) Mul1 expression may interfere with the balance between mitochondrial fusion and fission in sensory-innervated SKM cells; and (5) the mitochondrial downstream target AMPKα may be inhibited by IGF-1, and activation of AMPKα may block the effects of IGF-1 on the preservation of the contractility of sensory-innervated SKM cells. In the present study, a rat model of sensory protection was established to examine the potentiation effect of IGF-1 on sensory innervation in denervated muscles *in vivo*. To achieve sensory protection, we anastomosed the distal ending of the tibial nerve to the proximal ending of the sural nerve, which provided sensory innervation signal for distal SKM. To enhance the sensory innervation, exogenous IGF-1 was administrated in the sensory protection rat model. An *in vitro* sensory innervation model was also established by using dorsal root ganglion (DRG) explants and SKM cells to create a neuromuscular coculture. The enhanced effect of exogenous IGF-1 on the sensory innervation was detected. The mechanisms of the effects of IGF-1 on the sensory innervation *in vitro* were investigated by focusing on Akt, Mul1, and AMPKα, the downstream targets of IGF-1. The results of the present study will provide novel data that can be used when developing new approaches to improve the functional condition of denervated SKM cells by sensory protection combined with other enhancing factors.

## Results

### IGF-1 and sensory protection prevented denervated muscle atrophy and mitochondrial loss

**Figure 1:**
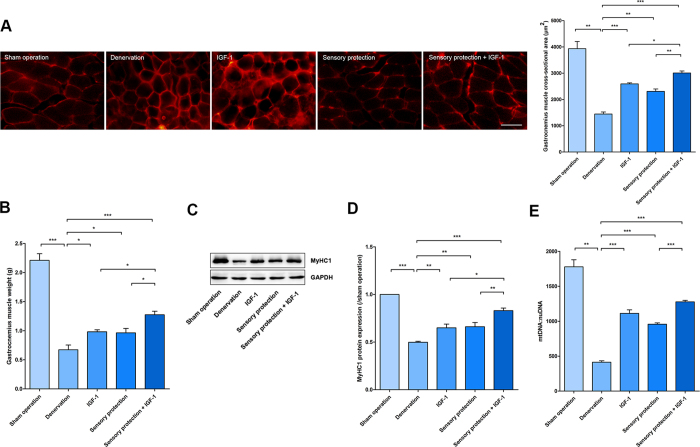
IGF-1 promoted the effects of sensory protection *in vivo*. (**A**) Cryosections of GAS muscles were stained with dystrophin. Representative images are shown. Scale bar = 50 μm. Mean CSA of GAS muscles in the sham operation, denervation, IGF-1, sensory protection and sensory protection + IGF-1 groups at the 8th week after surgery (n = 5). (**B**) Mean GAS muscle wet weight for the different treatments. (**C**) The total protein extracted from GAS muscle samples was immunoblotted with antibodies against MyHC1 and GAPDH. GAPDH was used as a loading control. (**D**) Quantification of MyHC1 protein levels (n = 5). (**E**) Quantification of the number of mitochondria in GAS muscle cells (n = 5). Bar graphs with error bars showing the mean ± SEM. *P < 0.05, **P < 0.01, ***P < 0.001.

After the different treatments were applied to the right hind limb of each group, gastrocnemius (GAS) muscle samples were collected at the 8th week post-surgery. The average area was measured by immunofluorescence labelling of dystrophin (Fig. 1A). The average cross-sectional area (CSA) and wet weight of GAS muscles increased in the IGF-1, sensory protection, and IGF-1+ sensory protection groups. The average area and weight in the IGF-1 and sensory protection groups was moderately higher than that in the denervated group. Furthermore, the IGF-1+ sensory protection group achieved a higher improvement than the groups that received IGF-1 or sensory protection alone (Fig. 1A,B). The contractile protein myosin heavy chain (MyHC) is an important mechanical component of myofilaments. MyHC alterations reflect the dynamic balance between protein synthesis and degradation and the functional status of SKM cells^26^,^27^. An obvious downregulation of MyHC1 has been demonstrated in denervated muscle. The MyHC1 expression levels of different groups corresponded to CSA and wet weight (Fig. 1C,D).

The MyHC1 downregulation was largely dependent on mitochondrial status^22^. The number of mitochondria, which may partially represent the functional status of the corresponding cell, was evaluated using the mtDNA per nuclear genome (mtDNA:nuDNA) ratio in the present study. The number of mitochondria in the sham operation, denervation, IGF-1, sensory protection and sensory protection + IGF-1 groups was 1779 ± 101, 414 ± 20, 1112 ± 51, 957 ± 19, and 1277 ± 21, respectively (Fig. 1E). This result demonstrated that sensory protection with or without IGF-1 prevented both the GAS muscle atrophy and mitochondria loss caused by denervation, and the combination of IGF-1 and sensory protection achieved the best effects.

### Immunofluorescence labelling to evaluate the morphology in the different treatment groups

**Figure 2:**
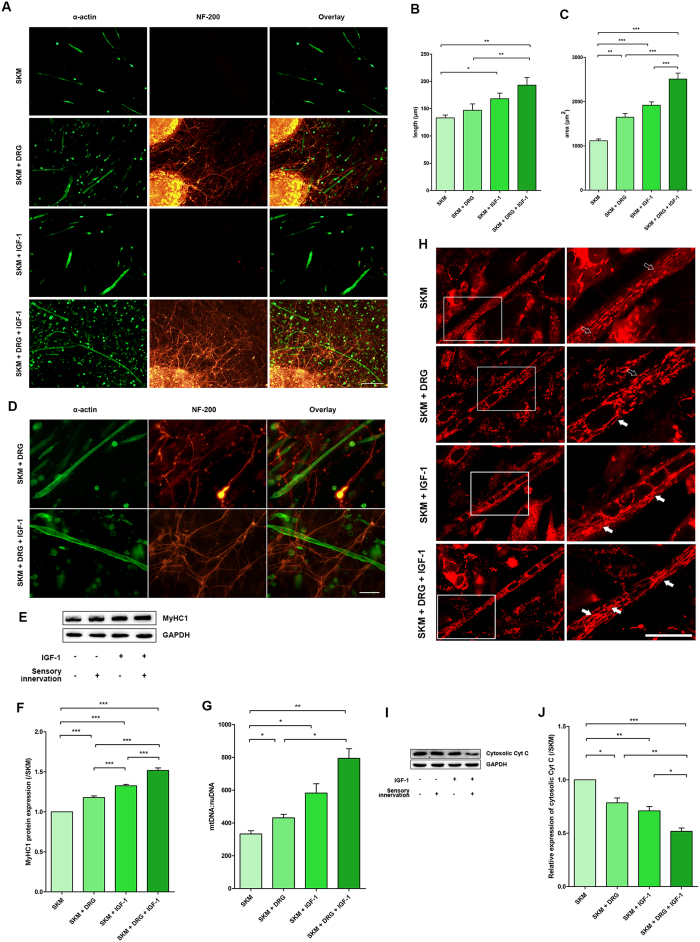
IGF-1 potentiated the effects of sensory innervation *in vitro*. (**A**) Double immunofluorescence labelling of α-actin (Green) and NF-200 (Red) indicated the SKM cell status in the SKM, SKM + DRG, SKM + IGF-1, and SKM + DRG + IGF-1 groups. Scale bar = 200 μm. (**B**) Quantification of the average length of each SKM cell (n = 5). (**C**) Quantification of the average area of each SKM cell (n = 5). (**D**) Double immunofluorescence labelling of α-actin (green) and NF-200 (red) showed dense networks of axons on the surface of SKM cells. Scale bar = 50 μm. (**E**) The total protein extracted from SKM cells was immunoblotted with antibodies against MyHC1 and GAPDH. GAPDH was used as a loading control. (**F**) Quantification of MyHC1 protein levels (n = 5). (**G**) Mitochondrial copy number in SKM cells (n = 5). (**H**) Mitochondrial morphology observed using MitoTracker Red staining in living SKM cells. The right column is an enlarged view of the box in the left column. The solid arrows in the zoomed area highlight the filamentous mitochondria, and the hollow arrows indicate the particulate mitochondria. Scale bar = 20 μm. (**I**) Cytosolic Cyt C protein immunoreactive bands. (**J**) Quantification of cytosolic Cyt C protein levels (n = 5). Bar graphs with error bars showing the mean ± SEM. *P < 0.05, **P < 0.01, ***P < 0.001.

To better control the experimental conditions, we modelled sensory innervated *in vitro* using SKM cells. The morphology of SKM cells and DRG neurons in different cultures with different treatments was observed by double immunofluorescence labelling of α-actin (for SKM cells) and NF-200 (for neurons) (Fig. 2A). The morphology of the SKM cells in cultures with different treatment conditions was similar. However, the average length and area of each SKM cell increased in the presence of DRG explants and/or IGF-1. Although there was no difference between SKM and SKM + DRG groups in the average length of each SKM cell, the average area was clearly higher in the SKM + DRG group. The average length and area in the SKM + IGF-1 group were both increased, and the average area in the SKM + DRG + IGF-1 group was even higher (Fig. 2B,C).

In the neuromuscular cocultures, DRG neurons sent axons across or terminating on the surface of SKM cells. The dense network of axons was observed on the surface of the SKM cells around the DRG explants. IGF-1 incubation could enhance formation of the axon network. Hence, a much denser network was observed on the surface of SKM cells after IGF-1 treatment (Fig. 2D).

### IGF-1 and sensory innervation promoted contractile protein expression, increased the number of mitochondria, prevented mitochondrial fission, and maintained mitochondrial integrity

SKM cells that are cultured alone *in vitro* may represent denervated SKM cells *in vivo*. SKM cells cultured with DRG explants *in vitro* may represent the SKM cells with sensory innervation *in vivo*. In the present study, IGF-1 administration promoted MyHC1 expression in SKM cells with or without sensory innervation *in vitro*. The SKM + DRG and SKM + IGF-1 groups both showed mild upregulation of the expression of MyHC1 content, with the highest elevation in the SKM + DRG + IGF-1 group, as indicated by comparison to the SKM group via Western blot analysis (Fig. 2E,F).

The average number of mitochondria in each SKM cell showed the same trend as the MyHC1 protein expression; IGF-1 incubation increased the number of mitochondria in each SKM cell in both SKM cells cultured alone and SKM cells with DRG explants. However, IGF-1 may have a large latent capacity to increase the number of mitochondria in SKM cells that received sensory innervation (Fig. 2G). This result suggested that IGF-1 might either potentiate the effect of sensory innervation by increasing the number of mitochondria or improve the responsiveness of SKM cells to sensory innervation.

The existence of a suitable balance between mitochondrial fission and fusion maintains the dynamic changes in the normal morphology and function of mitochondria. Excessive mitochondrial fission leads to mitochondrial loss and muscle atrophy. In the present study, mitochondrial fission was shown by MitoTracker Red staining for mitochondria in living cells. The accumulation of the probe is dependent on the membrane potential so the staining will provide images that can be used to evaluate the mitochondrial activity and the morphology. More filamentous and tubular or thread-like mitochondria were found in the SKM cells after sensory innervation and IGF-1 treatment, suggesting that IGF-1 could enhance the effects of sensory innervation, specifically the promotion of mitochondrial fusion and the inhibition of mitochondrial fission (Fig. 2H).

Cytochrome C (Cyt C) normally resides in the intermembrane space (IMS) of the mitochondria. The cytosolic Cyt C level represents mitochondrial integrity^28^. With the application of sensory innervation and IGF-1, cytosolic Cyt C levels decreased markedly, which demonstrated improvement of mitochondrial integrity (Fig. 2I,J).

### IGF-1 improved SKM cell status through phosphorylation of Akt in neuromuscular cocultures

**Figure 3:**
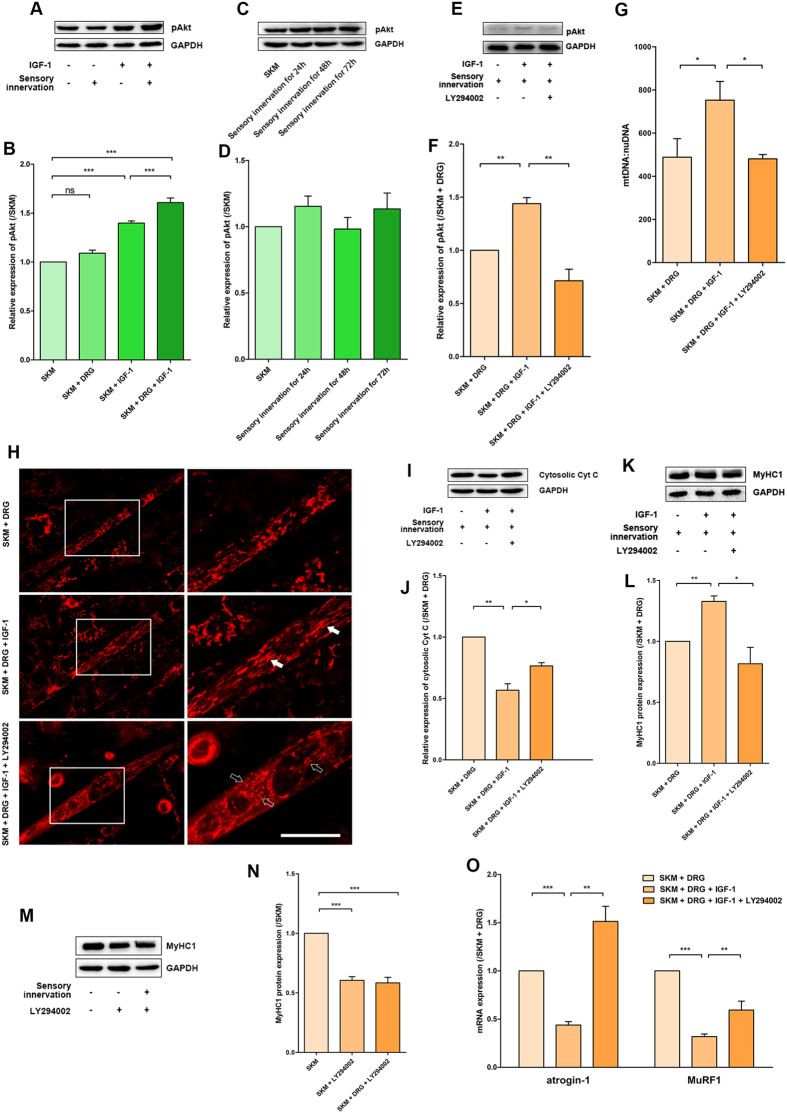
IGF-1 improved sensory-innervated SKM cell status by Akt phosphorylation. (**A**) Immunoreactive bands of phosphorylated Akt and GAPDH. (**B**) The pAkt protein expression was normalized to that of the SKM group and is shown as the fold change (n = 5). (**C**) Immunoreactive bands of phosphorylated Akt. (**D**) Quantification of pAkt protein levels at different time points (n = 5). (**E**) Immunoreactive bands of phosphorylated Akt and GAPDH in the SKM + DRG, SKM + DRG + IGF-1 and SKM + DRG + LY294002 + IGF-1 groups. (**F**) Quantification of pAkt protein levels with LY294002 administration (n = 5). (**G**) Quantitative analysis of mtDNA copy number per nuclear genome in SKM cells (n = 5). (**H**) MitoTracker Red staining for mitochondrial fission/fusion. The solid arrows and the hollow arrows indicate filamentous and particulate mitochondria, respectively. Scale bar = 20 μm. (**I**) Cytosolic Cyt C protein immunoreactive bands. (**J**) Quantification of cytosolic Cyt C protein levels (n = 5). (**K**) MyHC1 protein immunoreactive bands. (**L**) The expression of MyHC1 protein was normalized to that of the SKM + DRG group and is shown as the fold change (n = 5). (**M**) MyHC1 protein immunoreactive bands for exploring the effect of LY294002 to innervated SKM cells. (**N**) Quantification of MyHC1 protein levels (n = 5). (**O**) Levels of atrogin-1 and MuRF1 mRNA (n = 5). Bar graphs with error bars showing the mean ± SEM. *P < 0.05, **P < 0.01, ***P < 0.001.

Akt, a node in the IGF-1-related PI3K/Akt signalling pathway, plays a critical role in controlling cell survival. The multiple biological effects produced by IGF-1 could be blocked by inhibition of Akt phosphorylation. In the present study, the levels of phosphorylated Akt (pAkt) were measured in different conditions after IGF-1 treatment for 1 hour. The pAkt levels in the SKM + IGF-1 group increased moderately; additionally, the highest activation level was observed in the SKM + DRG + IGF-1 group. However, there was no difference in the phosphorylation of Akt between the SKM and neuromuscular cocultures (Fig. 3A,B). To further exclude a different timing of activation, we tested Akt phosphorylation level at 24 h, 48 h, and 72 h after sensory innervation, and there was no difference compared with SKM group (Fig. 3C,D). This result showed that sensory innervation did not activate the Akt pathway and might protect SKM cells in other ways. However, IGF-1 showed a more obvious promotion effect on the Akt phosphorylation of SKM cells in neuromuscular cocultures.

To further clarify the role of the PI3K/Akt signalling pathway in improving SKM cell status by Akt phosphorylation in IGF-1-treated neuromuscular cocultures, the PI3K inhibitor LY294002 (10 μmol/L) was applied to the neuromuscular cocultures 30 minutes before IGF-1 treatment at 2 days of coculture. One hour after IGF-1 treatment, the pAkt levels suggested that the administration of the PI3K inhibitor LY294002 blocked the effects of IGF-1 on Akt phosphorylation in the SKM cells in neuromuscular cocultures (Fig. 3E,F).

After the above treatment for 2 days, the mtDNA:nuDNA ratio in the SKM cells demonstrated that LY294002-induced Akt phosphorylation inhibition reversed the increase in mitochondria (Fig. 3G). The MitoTracker Red labelling showed more particulate or discontinuous mitochondria in the LY-cotreatment group (Fig. 3H). The cytosolic Cyt C levels noted LY294002 administration reversed the improvement of mitochondrial integrity (Fig. 3I,J). The mitochondrial patterns indicated that Akt phosphorylation participated in mitochondrial fusion.

Furthermore, IGF-1 administration failed to increase the MyHC1 protein levels in the SKM + DRG + LY294002 + IGF-1 group (Fig. 3K,L). To clarify whether LY294002 inhibited the effect of sensory innervation, LY294002 was applied to SKM cell culture and neuromuscular coculture of DRG and SKM cells, respectively. MyHC1 levels were paralleled decreased in SKM alone or sensory innervated SKM after LY294002 inhibition. These results implied that LY294002 could not inhibit the rescue effect of DRG sensory innervation on SKM (Fig. 3M,N). The mRNA levels of atrogin-1 and MuRF1, the E3 ubiquitin ligases that direct polyubiquitinated skeletal muscle proteins to proteolysis in the ubiquitin proteasome system (UPS), were increased in the SKM + DRG + LY294002 + IGF-1 group (Fig. 3O). These results suggested that IGF-1 treatment decreased the atrogin-1 mRNA and MuRF1 mRNA levels and finally promoted MyHC1 protein expression by Akt phosphorylation in neuromuscular cocultures.

### Mul1 overexpression promoted mitochondrial fission, decreased the mtDNA content, and destructed mitochondrial integrity

**Figure 4:**
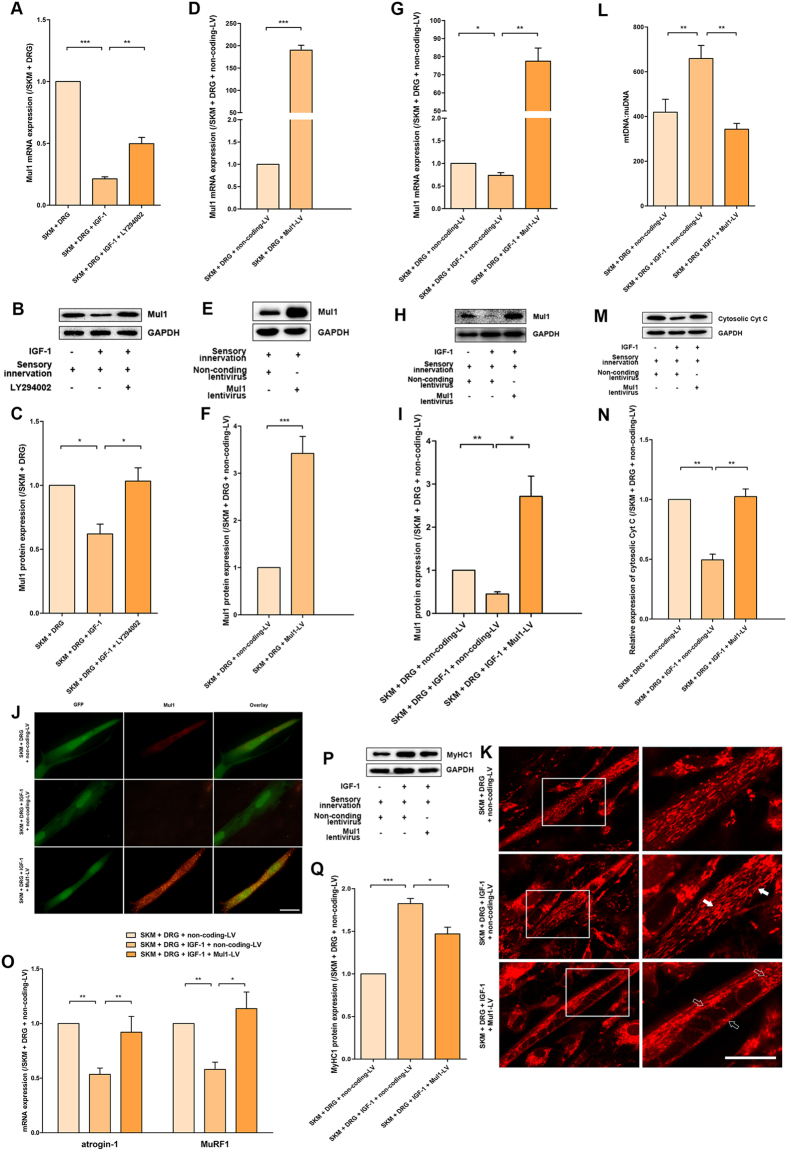
IGF-1 promoted the effects of sensory innervation by suppressing Mul1 expression. (**A**) Quantification of Mul1 mRNA for LY294002 administration (n = 5). (**B**) Immunoreactive bands of Mul1 and GAPDH after LY294002 treatment. (**C**) Quantification of Mul1 protein levels (n = 5). (**D**) Mul1 mRNA levels in the SKM + DRG (non-coding lentivirus) and SKM + DRG (Mul1 lentivirus) groups (n = 5). (**E**) Mul1 protein immunoreactive bands after lentivirus transfection in the above two groups. (**F**) Quantification of Mul1 protein levels (n = 5). (**G**) Mul1 mRNA levels in the SKM + DRG (non-coding lentivirus), SKM + DRG (non-coding lentivirus) + IGF-1 and SKM + DRG (Mul1 lentivirus) +IGF-1 groups (n = 5). (**H**) Mul1 protein immunoreactive bands after lentivirus transfection. (**I**) Quantification of Mul1 protein levels after lentivirus transfection (n = 5). (**J**) The immunofluorescence staining to confirm the validity of Mul1 lentivirus transfection. GFP (green) shows the successful infection of the lentivirus, and the Mul1 (red) labelling indicates the expression of Mul1 protein. (**K**) MitoTracker Red staining for mitochondrial fission/fusion of SKM cells infected by the lentivirus. Scale bar = 20 μm. (**L**) Quantitative analysis of mtDNA copy number per nuclear genome in SKM cells (n = 5). (**M**) Cytosolic Cyt C protein immunoreactive bands after lentivirus transfection. (**N**) Quantification of cytosolic Cyt C protein levels (n = 5). (**O**) Levels of atrogin-1 and MuRF1 mRNA (n = 5). (**P**) Immunoreactive bands of MyHC1 and GAPDH. (**Q**) Quantitated MyHC1 protein abundance (n = 5). Bar graphs with error bars showing the mean ± SEM. *P < 0.05, **P < 0.01, ***P < 0.001.

Mul1 is a key regulator of SKM mitochondrial fission. The expression of Mul1 and its mRNA could be inhibited by the presence of IGF-1. The effects of IGF-1 on the inhibition of Mul1 expression could be blocked by the PI3K inhibitor LY294002 (Fig. 4A–C).

To further investigate whether Mul1 gene overexpression could counteract the effect of IGF-1, the rat Mul1 gene was inserted into a lentiviral vector, which was transfected into SKM cells. The validity of Mul1 lentivirus transfection was confirmed by the much higher levels of mRNA and protein expression in Mul1 lentivirus-infected SKM cells even in the presence of sensory innervation (Fig. 4D–F). Though the overexpression of Mul1 might be interfered by the presence of IGF-1, Mul1 mRNA and protein expression level also increased significantly (Fig. 4G–I). Prominent dotted Mul1 fluorescence labelling *in situ* was also observed only in Mul1 lentivirus-infected SKM cells, whereas non-coding lentivirus-infected SKM cells showed only weak and diffuse Mul1 fluorescence (Fig. 4J).

With the successfully established Mul1 expression culture system, the effects of Mul1 on its downstream target or signalling cascade were tested. MitoTracker Red labelling showed disrupted and fragmented mitochondria in the entire cell, demonstrating a remarkable trend toward mitochondrial fission with Mul1 overexpression (Fig. 4K). The mtDNA:nuDNA ratio in SKM cells in the SKM + DRG (non-coding lentivirus), SKM + DRG (non-coding lentivirus) + IGF-1, and SKM + DRG (Mul1 lentivirus) + IGF-1 groups was 419 ± 58, 659 ± 59, and 343 ± 26, respectively (Fig. 4L). Cytosolic Cyt C level was upregulated by Mul1 expression (Fig. 4M,N).

The atrogin-1 and MuRF1 mRNA levels showed evident increases after Mul1 overexpression between the SKM + DRG + IGF-1 groups with or without Mul1 overexpression (Fig. 4O). Consistent with the atrogin-1 and MuRF1 expression results, the MyHC1 level in the SKM + DRG (Mul1 lentivirus) + IGF-1 group was higher than that in the SKM + DRG (non-coding lentivirus) + IGF-1 group (Fig. 4P,Q). These results suggested that Mul1 overexpression could abolish the effects of IGF-1 on mtDNA level elevation, mitochondrial fission inhibition, atrogin-1 and MuRF1 mRNA expression inhibition, and MyHC1 protein expression promotion.

### AMPKα activation inhibited MyHC1 through upregulation of atrogin-1 and MuRF1

To further clarify the mechanisms of IGF-1 signalling for the improvement of myocyte status by modulating the balance of mitochondrial fission/fusion in neuromuscular cocultures, AMPKα was measured as a downstream target of mitochondrial energy production. The effects of AMPKα activation on contractile protein expression and its mechanisms were investigated. The activation of AMPKα turns on the switch that promotes ATP generation under conditions of metabolic stress that deplete ATP while also increasing ADP and AMP and repressing energy-consuming processes. AMPKα activation also prevents adipocyte differentiation and the expression of lipid biosynthetic enzymes, such as acetyl-CoA carboxylase (ACC), which converts acetyl-CoA to malonyl-CoA and is a pivotal enzyme of the lipogenic pathway^29^.

**Figure 5:**
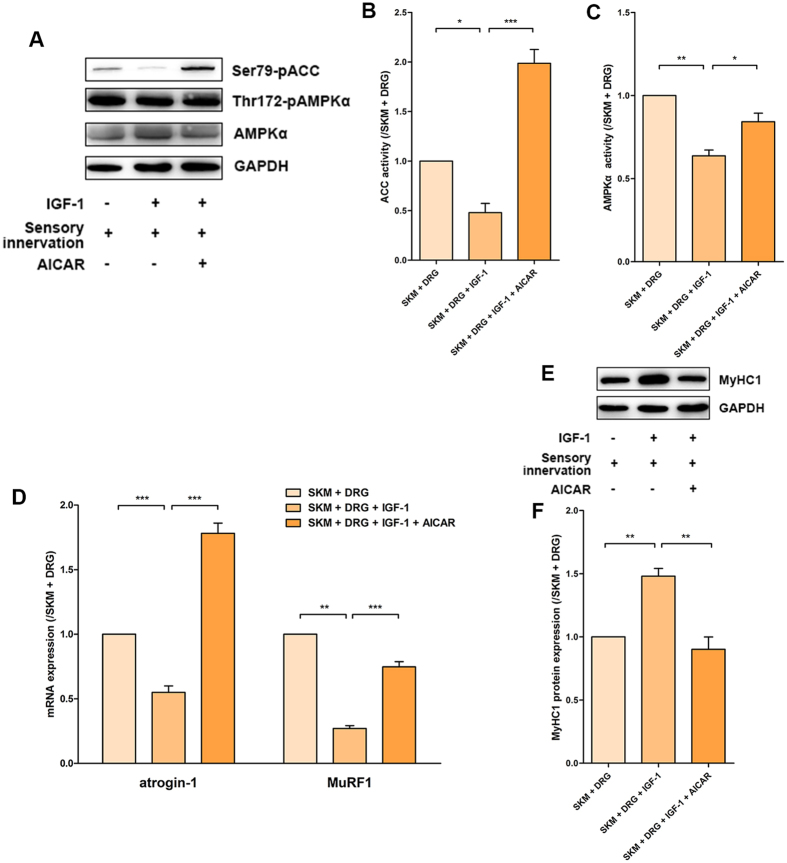
AMPKα activation of sensory-innervated SKM cells was modulating by IGF-1. (**A**) Immunoreactive bands of pACC, pAMPKα, AMPKα and GAPDH for the 6-hour AICAR treatment. (**B**) Quantification of the pACC levels (n = 5). (**C**) Quantification of the pAMPKα levels (n = 5). (**D**) Levels of atrogin-1 and MuRF1 mRNA for the 2-day AICAR treatment (n = 5). (**E**) MyHC1 immunoreactive bands for the 2-day AICAR treatment. (**F**) Quantification of the MyHC1 protein levels (n = 5). Bar graphs with error bars showing the mean ± SEM. *P < 0.05, **P < 0.01, ***P < 0.001.

5-aminoimidazole-4-carboxamide-1-D-ribonucleoside (AICAR) was commonly used to activate AMPK directly without changing cellular ATP, ADP, and AMP concentrations. In the present study, the lower pAMPKα and pACC levels indicated suppression of AMPKα in the SKM + DRG + IGF-1 group compared with the SKM + DRG group, but 6 hours of AICAR treatment induced AMPKα activation in the SKM + DRG + AICAR + IGF-1 group that reversed the descending trend (Fig. 5A–C). After treatment with AICAR for 2 days, the mRNA levels of atrogin-1 and MuRF1 were much higher in the SKM + DRG + AICAR + IGF-1 group than in the SKM + DRG + IGF-1 group (Fig. 5D). Correspondingly, AICAR administration decreased the MyHC1 levels in the SKM + DRG + AICAR + IGF-1 group (Fig. 5E,F). These results suggested that AMPKα activation in the neuromuscular coculture system could counteract the effects of IGF-1 on AMPKα inhibition, atrogin-1 mRNA and MuRF1 mRNA expression inhibition, and MyHC1 protein expression promotion.

## Discussion

The recovery of contractile function of long-term denervated SKM cells is so difficult that long-term denervated SKM cells cannot even recover after motor nerve reinnervation is re-established because the denervation time is too long and the contractility has been lost. Sensory protection has been suggested as a promising technique when motor nerve repair is delayed or when injury occurs at a proximal point^4^. However, sensory protection is not sufficient to improve the functional recovery of long-term denervated SKM cells. Hence, the use of pharmacological intervention to enhance the efficacy of sensory protection and finally attain the goal of functional recovery improvement may be a valid therapeutic strategy. In the present study, the effects and the mechanisms of the IGF-1-induced improvement of the SKM status were investigated using a sensory protection model *in vivo* and a specific neuromuscular coculture system *in vitro* and through interference with several targets in the IGF-1 signalling cascade. The results showed that IGF-1 further improved the sensory-innervated SKM cell status. IGF-1-induced Akt phosphorylation is a key step in preserving the contractility of sensory-innervated SKM cells. The expression of Mul1, a mitochondrial outer-membrane protein, was suppressed by IGF-1-induced Akt phosphorylation. Overexpression of Mul1 blocked the effects of IGF-1 that preserved the contractility of sensory-innervated SKM cells. Mul1 overexpression interfered with the balance between mitochondrial fusion and fission in sensory-innervated SKM cells. IGF-1 could inhibit the mitochondrial downstream target AMPKα. Activation of AMPKα blocked the effects of IGF-1 that preserved the contractility of sensory-innervated SKM cells.

First, IGF-1 and sensory protection was induced in a denervation animal model. The results showed that IGF-1 enhanced the effects of sensory protection in denervated muscle, which included staving off atrophy and preventing mitochondrial loss. Furthermore, the effects of IGF-1 on SKM cells with or without sensory innervation were determined using SKM cell culture alone or neuromuscular coculture of DRG explants and SKM cells. IGF-1 had more obvious effects that improved the SKM cell status in neuromuscular coculture. These results prompted us to investigate the mechanisms underlying the IGF-1-induced improvement of SKM cell status using neuromuscular cocultures of DRG explants and SKM cells with IGF-1 treatment.

Due to the importance of the Akt signalling cascade in the IGF-1 signalling system^30^,^31^,^32^ and its critical actions on SKM cells^13^, we blocked Akt phosphorylation by administering the PI3K inhibitor LY294002 in the neuromuscular cocultures of DRG explants and SKM cells. The effects of IGF-1 on the inhibition of mitochondrial fission, increase in mtDNA levels, promotion of mitochondrial integrity, decrease in atrogin-1 mRNA and MuRF1 mRNA levels, and promotion of MyHC1 protein expression could be blocked by LY294002 administration in the neuromuscular cocultures. Even sensory innervation cannot activate Akt phosphorylation at different time points, LY294002 was capable to inhibit the basal Akt phosphorylation levels and as a result suppress the MyHC1 expression. These results suggested that Akt is a key node for improving the status of SKM cells that receive sensory innervation *in vitro*.

These results indicated that Akt phosphorylation improved the SKM cell status mainly by modulating the balance between mitochondrial fusion and fission. Accordingly, we focussed on another target, Mul1, a factor that regulates dynamic mitochondrial alterations. Because the Akt phosphorylation induced by IGF-1 inhibited Mul1 expression and then induced a high level of mtDNA, we further measured the effects of Mul1 in these processes by transfecting SKM cells with a Mul1-overexpression lentivirus. Intriguingly, Mul1 overexpression could disrupt the effects of IGF-1 on the modulation of the balance between mitochondrial fusion and fission and then interfere with the contractile protein expression of SKM cells. These results implied that Mul1 is an important factor for targeting mitochondrial homeostasis of SKM cells that receive sensory innervation.

**Figure 6:**
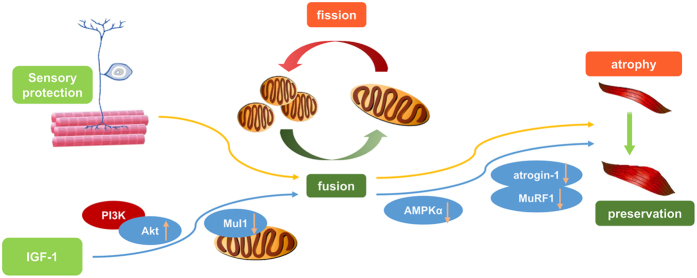
Schema of the mechanism of IGF-1 on enhancing sensory innervation efficacy. IGF-1-induced Akt phosphorylation suppressed Mul1 expression, restored the balance between mitochondrial fusion and fission, and inhibited activation of AMPKα to preserve the intrinsic contractility of sensory-innervated SKM cells.

All the above results showed that IGF-1, through Akt activation and inhibition of Mul1 expression, elevated mtDNA levels and achieved a better pattern of mitochondrial homeostasis in SKM cells that received sensory innervation (Fig. 6). However, the link between mitochondria and contractile proteins still needs to be clarified. Because mitochondria are the primary energy-producing organelles, it is presumed that more energy production is beneficial for maintaining protein structure. The relevance of mitochondria to functional proteins might be found in previous studies on the cellular energy sensor AMPKα^21^,^23^,^24^. Therefore, the effects of the mitochondrial downstream target AMPKα were also further determined using the administration of the AMPKα activator AICAR in the neuromuscular cocultures. Activation of AMPKα by AICAR counteracted the effects of IGF-1 on AMPKα inhibition, atrogin-1 mRNA and MuRF1 mRNA expression inhibition, and MyHC1 protein expression promotion. These results implied that the mitochondrial downstream target AMPKα might be a critical link between mitochondrial homeostasis and contractile protein synthesis. Inhibition of AMPKα might be beneficial for the contractile protein synthesis and functional recovery of the SKM cells that received IGF-1 and sensory innervation.

In conclusion, the results of the present study, which used an *in vivo* sensory protection model and an *in vitro* model of SKM cells that received sensory innervation, revealed that IGF-1 improves myocyte status through targeting mitochondrial upstream targets, Akt and Mul1, and the mitochondrial downstream target AMPKα (Fig. 6). The main function of this pathway may lead to IGF-1-induced Akt phosphorylation and Mul1 expression inhibition, followed by better pattern of mitochondrial homeostasis, which results in an increase in MyHC1 synthesis through inhibition of atrogin-1 and MuRF1 expression. These data provided novel evidence that can be used to develop new approaches to improve the functional condition of long-term denervated SKM cells by interfering with the IGF-1 signalling system and its function in modulating the balance between mitochondrial fusion and fission.

## Methods

### Ethics statement

All animals received good care in accordance with the National Institutes of Health Guide for the Care and Use of Laboratory Animals (eighth edition, 2010). All protocols described in this article were approved prior to the study and reviewed afterwards by the Ethical Committee for Animal Experimentation of Shandong University. All surgery procedures for experimental animals were performed under anaesthesia with 3.0% pentobarbital sodium (1 ml/kg, body weight) by intraperitoneal injection (i.p.), and we minimized suffering of the laboratory animals to the best of our ability.

### Animal models

Twenty-five adult male Wistar rats, weighing approximately 260 g ± 10 g, were randomly assigned to sham operation, denervation, IGF-1, sensory protection, and IGF-1+ sensory protection groups (5 rats per group). The surgical operation was performed on the right hind limb of each animal. In brief, for the sham operation group, a longitudinal incision exposed the trifurcation of the sciatic nerve to free the tibial, peroneal and sural nerves. For the denervation group, the same incision was made, and the tibial nerve was transected. The proximal stumps were ligated and sewn into a cap to prevent regenerated fibres from reconnecting the distal stumps. For the sensory protection group and the IGF-1+ sensory protection group, the tibial nerve and sural nerve were transected, and the distal ending of the tibial nerve was anastomosed to the proximal ending of the sural nerve in an end-to-end fashion. The proximal stump of the tibial nerve was also ligated and sewn into a cap. For IGF-1 administration in the IGF-1 group and the IGF-1+ sensory protection group, GAS muscles of the right hind limbs were injected with 10 μg of IGF-1 (PreproTech) every 3 days following the surgery until 8 weeks.

### Muscle wet weight and CSA measurement

GAS muscle samples were collected at week 8 after surgery. Muscle wet weight was recorded immediately. Transverse muscle sections of 20-μm thickness were obtained by cryosectioning. The standard procedure was used to perform the immunostaining with mouse-anti-dystrophin (1:200, Abcam). Slides were maintained with anti-fade mounting medium (Santa Cruz Biotechnology). Cross sections were examined in a blinded procedure to quantify individual fibre CSAs. Image-Pro Plus software (version 6.0.0.260) was used to measure individual fibre CSAs.

### Cell culture preparations

Primary SKM cell culture preparations utilized muscles from the hind limbs of newborn rats. The primary cultured myocytes purified by differential attachment were grown in Dulbecco’s modified Eagle medium with F-12 supplement (DMEM/F-12) (Gibco) containing 20% foetal bovine serum (Gibco) and 1% penicillin/streptomycin (Gibco), which promotes myocyte proliferation and is termed SKM medium. The SKM cells were incubated at 37 °C in a 5% CO_2_ incubator.

At 2 days of SKM cell culture as described above, organotypic DRG cultures were prepared to establish the sensory innervation model of DRG explants and dissociated SKM cells. Briefly, bilateral DRGs were taken from each embryo at embryonic day 15 (E15) and placed in 24-well clusters containing single-layered myocytes in DMEM/F-12 supplemented with 5% foetal bovine serum, 2% B-27 supplement (Gibco), L-glutamine (0.1 mg/ml, Sigma) and 1% penicillin/streptomycin to maintain the DRG cells and myotube fusion, which is referred to as coculture medium.

For real-time PCR and Western blot assays of SKM cells, the DRG explants in the cocultures were removed prior to harvesting the SKM cells.

### Administration of different reagents and transfection with lentivirus *in vitro*

The SKM cell cultures and sensory innervation cultures were treated as follows: (1) SKM group: The SKM cells were cultured for 2 days with SKM medium, and the culture continued for another 4 days with coculture medium; (2) SKM + DRG group: SKM cells were cultured for 2 days and were then cocultured for another 4 days with DRG explants; (3) SKM + IGF-1 group: The SKM cells were cultured for 2 days with SKM medium, 2 days with coculture medium, and another 2 days with coculture medium containing IGF-1 (20 nmol/L); and (4) Coculture + IGF-1 group: The SKM cells were cultured for 2 days in SKM medium and another 4 days with coculture medium with DRG explants; IGF-1 (20 nmol/L) was applied in the last 2 days. After processing for the live monitoring of mitochondrial morphology, real-time PCR and Western blot assays of the SKM cells, the SKM + DRG + IGF-1 group was treated with either the PI3K inhibitor LY294002 (10 μmol/L, Invitrogen), Mul1 overexpression lentivirus, or the AMPKα activator AICAR (1 mmol/L, Abcam) for further exploration of the mechanisms underlying the effects of IGF-1 on SKM cells in neuromuscular cocultures.

### DNA isolation and mtDNA quantitation

Total DNA extraction was performed with a DNA kit (Tiangen Biotech). The quantitation of the mtDNA per nuclear genome used in this study was performed as previously described^33^,^34^. In brief, with real-time PCR analysis, the NADH dehydrogenase subunit 1 (ND1), which represents the mtDNA, was normalized to the Pecam gene on chromosome 6, which represents nuDNA. The synthetic oligonucleotide primer sequences for ND1 are 5′-GTC CTC CTA ATA AGC GGC TCC T-3′ (coding sense) and 5′-GAA TGG TCC TGC GGC GTA TTC-3′ (coding antisense), and the sequences of Pecam are 5′-CTA TGG CGG ACA CCT TCC TG-3′ (coding sense) and 5′-TTC TAG GCC TTG GGT GGT CT-3′ (coding antisense). Real-time PCR was performed using SYBR Green dye (Thermo Scientific) with the Eppendorf Realplex PCR system. The relative copy number differences were quantified by analysing the difference in threshold amplification between mtDNA and nuDNA (ΔΔCt method).

### MitoTracker Red labelling for mitochondria *in vitro*

For measuring the MitoTracker Red staining, myotubes were incubated with MitoTracker CMX-Ros Red (MTRed) (Invitrogen). Images of myotube mitochondria were captured using fluorescence microscopy. Image contrast and brightness manipulations were performed using Olympus CellSens Dimension software (version 1.6).

### Real-time PCR analysis

Total RNA was extracted using the RNA Fast200 kit (Fastagen). cDNA was synthesized using a cDNA synthesis kit (Thermo Scientific). Real-time PCR was performed using SYBR Green dye according to the manufacturer’s instructions, and amplification was performed with synthetic oligonucleotide primer sequences, including the following: Mul1 5′-CCG CCG TAC TGT ACT CCA TAT-3′ (coding sense) and 5′-ACT GGC TGT TGA GCG TTT CT-3′ (coding antisense), atrogin-1 5′-GAT GGA TTG GAA GAA GAT G-3′ (coding sense) and 5′-TGA AAG TGA GAC GGA GCA G-3′ (coding antisense), MuRF1 5′-CAG GAA TGC TCC AGT CGG-3′ (coding sense) and 5′-CGT CCA GGA TGG CGT AG-3′ (coding antisense), and glyceraldehyde 3-phosphate dehydrogenase (GAPDH) 5′-GGC ACA GTC AAG GCT GAG AAT G-3′ (coding sense) and 5′-ATG GTG GTG AAG ACG CCA GTA-3′ (coding antisense). A comparative cycle of the threshold fluorescence (Ct) method was used, and the relative amount of the target gene transcript was normalized to that of GAPDH using the 2^−ΔΔCt^ method based on previous studies.

### Immunofluorescence labelling *in vitro*

SKM cell cultures and neuromuscular cocultures with different treatments were processed for immunofluorescence labelling. Cells on the coverslips were fixed in 4% paraformaldehyde (pH 7.4) for 40 minutes, permeated with 0.3% Triton X-100-PBS solution for 1 hour, blocked with 10% normal goat serum, and incubated with rabbit anti-NF-200 (1:1,000, Abcam), mouse anti-α-actin (1:500, Abcam), or rabbit anti-Mul1 (1:500, Sigma) overnight at 4 °C, followed by incubation with goat anti-rabbit conjugated to TRITC (1:200, Abcam) or goat anti-mouse conjugated to FITC (1:200, Abcam). Coverslips were kept on object slides with anti-fade mounting medium. For quantitative morphological analysis, the length and area of SKM cells *in vitro* were measured using Image-Pro plus software.

### Western blot assay

Protein abundance and phosphorylation were determined using a Western blot assay following standard procedures. Total proteins were lysed in RIPA lysis buffer. Isolation of cytosolic proteins was performed using the Mitochondria/cytosol Fractionation Kit (Beyotime). In brief, total protein was separated in SDS-PAGE and transferred to nitrocellulose (NC) membranes, and the following primary antibodies were used: mouse anti-MyHC1 monoclonal IgG (1:2,000, Upstate), rabbit anti-pAkt monoclonal IgG (1:1,000, Cell Signaling Technology), rabbit anti-Mul1 polyclonal IgG (1:500, Abcam), rabbit anti-pACC polyclonal IgG (1:1,000, Cell Signaling Technology), rabbit anti-pAMPKα monoclonal IgG (1:1,000, Cell Signaling Technology), rabbit anti-AMPKα monoclonal IgG (1:1,000, Cell Signaling Technology), mouse anti-Cyt C monoclonal IgG (1:1000, Abcam), and mouse anti-GAPDH monoclonal IgG (1:1,000, Goodhere). After incubation with goat anti-rabbit IgG-HRP (1:3,000, Santa Cruz Biotechnology) or goat anti-mouse IgG-HRP (1:3,000, Santa Cruz Biotechnology), immunoreactive bands were visualized with an ECL Western blotting detection kit (Millipore) using a FluorChem E System (Protein Simple Inc.). The images were analysed quantitatively using AlphaView 3.4.0 image analysis software. The results of the Western blot assay are presented as the ratio of each protein level to the corresponding control.

### Statistical analysis

All data are reported as the mean ± SEM. All data were processed to verify normality. The data with an abnormal distribution were analysed with a non-parametric test. If the data were normally distributed, the statistical analysis included a one-way analysis of variance followed by a least significant difference (LSD) test (homogeneity of variance) or Dunnett’s T3 test (heterogeneity of variance) to compare the differences among the various groups or a two independent sample t-test to compare the difference between two groups and analysed using SPSS (version 20.0.0) software. A *P* value < 0.05 was used to delineate significance for analysis of all results.

## Additional Information

**How to cite this article:** Ding, Y. *et al*. IGF-1 potentiates sensory innervation signalling by modulating the mitochondrial fission/fusion balance. *Sci. Rep.* **7**, 43949; doi: 10.1038/srep43949 (2017).

**Publisher's note:** Springer Nature remains neutral with regard to jurisdictional claims in published maps and institutional affiliations.
